# In Silico Comparative Transcriptome Analysis of Two Color Morphs of the Common Coral Trout (*Plectropomus Leopardus*)

**DOI:** 10.1371/journal.pone.0145868

**Published:** 2015-12-29

**Authors:** Le Wang, Cuiping Yu, Liang Guo, Haoran Lin, Zining Meng

**Affiliations:** State Key Laboratory of Biocontrol, Institute of Aquatic Economic Animals and the Guangdong Province Key Laboratory for Aquatic Economic Animals, School of Life Sciences, Sun Yat-sen University, Guangzhou, 510275, China; The Ohio State University, UNITED STATES

## Abstract

The common coral trout is one species of major importance in commercial fisheries and aquaculture. Recently, two different color morphs of *Plectropomus leopardus* were discovered and the biological importance of the color difference is unknown. Since coral trout species are poorly characterized at the molecular level, we undertook the transcriptomic characterization of the two color morphs, one black and one red coral trout, using Illumina next generation sequencing technologies. The study produced 55162966 and 54588952 paired-end reads, for black and red trout, respectively. *De novo* transcriptome assembly generated 95367 and 99424 unique sequences in black and red trout, respectively, with 88813 sequences shared between them. Approximately 50% of both trancriptomes were functionally annotated by BLAST searches against protein databases. The two trancriptomes were enriched into 25 functional categories and showed similar profiles of Gene Ontology category compositions. 34110 unigenes were grouped into 259 KEGG pathways. Moreover, we identified 14649 simple sequence repeats (SSRs) and designed primers for potential application. We also discovered 130524 putative single nucleotide polymorphisms (SNPs) in the two transcriptomes, supplying potential genomic resources for the coral trout species. In addition, we identified 936 fast-evolving genes and 165 candidate genes under positive selection between the two color morphs. Finally, 38 candidate genes underlying the mechanism of color and pigmentation were also isolated. This study presents the first transcriptome resources for the common coral trout and provides basic information for the development of genomic tools for the identification, conservation, and understanding of the speciation and local adaptation of coral reef fish species.

## Introduction

The high diversity of tropical coral-reef fish communities has been of great interest for ecological and evolutionary studies on cryptic diversity and molecular taxonomy [1, 2]. Coral reef fish, such as coral trout (*Plectropomus spp*.), in particular, exhibit high levels of phenotypic plasticity in skin color and pigmentation, body shape and size, fin morphology, as well as behavioral and ecological activities [3–5]. These characteristics of coral trout provide a unique model for studying the mechanism of speciation and local adaptation in diverse and complex coral-reef environments [6].

The common coral trout or leopard coral grouper (*Plectropomus leopardus*) is one such coral trout belonging to the Serranidae family [7]. Coral trout species are of great economic value as both precious marine food fish and ornamental fish of beautiful skin color [7, 8]. This coral trout is mainly distributed in the Western Pacific regions from southern Japan southward to Australia and eastward to the Caroline Islands [7]. Mitochondrial DNA distinguishes the two color morphs of the common coral trout (bright red vs dark red; both with small blue spots), according to work reported in 2013 and 2014 [9–11]. Due to its high economic value, the wild populations of this coral trout have been overexploited and considered threatened by IUCN [12]. Previous studies on the common coral trout were mainly focused on conservation ecology [13, 14], reproductive physiology [15, 16] and molecular classification [17, 18] as well as larval behavior and early life history [19, 20]. Studies on the exploitation of the genetic resources of this species were only focused on the development of microsatellite markers [21]. Thus, the genomic resources of this species are still scarce till now, which greatly limit the genetic studies and genetic conservation of this species.

The next generation sequencing technologies (NGS) have significantly reduced the cost of obtaining a large amount of sequence data, driving the quick development of whole genome sequencing and transcriptome sequencing (RNA-seq) for both model and nonmodel species [22]. At present, RNA-seq has been widely used to explore the whole transcriptomes of non-model fish species without reference genome information [23–25]. However, there are still no transcriptome sequencing studies on the common coral trout and its related species.

In order to obtain a comprehensive transcriptome dataset of this species, we sequenced two different color morphs of the common coral trout, one bright-red (red trout) and another dark-red (black trout), using Illunima NGS technology, and analyzed the transcriptomes in silico using various bioinformatics tools. Our aims were to (i) obtain high-quality transcriptome assemblies and functional annotations of the two color morphs of the common coral trout; (ii) discover a large number of SSR and SNP markers within and between the two color morphs; (iii) identify a group of fast-evolving genes between the two color morphs and also candidate genes involved in the formation of skin color and pigmentation. Above all, the two transcriptomes would be of great value in assisting gene discovery, functional genomics, population genetics, and future genome projects for coral fish species; the transcriptomic data could potentially help in understanding the mechanisms of speciation and adaptive evolution between the two different color morphs.

## Materials and Methods

### Sample collection and RNA extraction

The two color morphs of common coral trout were brought from Guangzhou Huangsha fish market in China, and were initially collected from the South China Sea through commercial fishing. The two fish were captured from the Dongsha islands of the South China Sea during one fishing activity. The body weight and total length for the red trout were 798.5 g and 39.3 cm, respectively, while the black trout were 910.6 g and 43.9 cm, respectively. Both of the two fish were female and of the similar age of about two years old judging by the body weight and total length. The fish were cultured in circulating seawater in laboratory at 30°C for one week and were fed twice daily before experiment. Fish were anesthetized with MS222 and sacrificed by decapitation for tissue dissection. To obtain the whole transcriptome profile, nine tissues including brain, pituitary, liver, gonad, head kidney, spleen, muscle and skin were sampled. The tissues were immediately frozen in liquid nitrogen after dissection and were then stored at -80°C until RNA extraction. All animal experiments were conducted in accordance with the guidelines and approval of the respective Animal Research and Ethics Committees of Sun Yat-Sen University. Total RNA was extracted from each tissue using Trizol reagent followed by digestion with RNase free DNase I (New England Biolabs) following the manufacturers’ protocol. RNA quality and concentration of each sample was measured using RNA Nano Bioanalysis chip on an Agilent 2100 Bioanalyzer (Agilent Technologies).

### Library construction and Next generation sequencing

Equal amount of RNA of each tissue from one fish was pooled together for library construction. Sequencing libraries were prepared using Illumina TruSeq RNA sample preparation kit (Illumina) for paired-end sequencing of 2×100bp using Illumina HiSeq^TM^2000 (Illumina). Library construction and paired-end sequencing were performed in a single lane by Beijing Genomics Institute (BGI), Shenzhen, China.

### Transcriptome assembly and functional Annotation

We firstly filtered the raw sequencing reads before carrying out transcriptome assembly. The raw sequencing reads were cleaned by removing adapters, low-quality tags with PHRED-like scores of less than 20 and reads with Ns and shorter than 35 bp after trimming using NGSQCToolkit (v2.3) [26]. *De novo* assembly of transcriptome was performed using Trinity [27].

The obtained unigenes were annotated using homology search (BLASTX) [28] with E-value cutoff of 10^−6^ against NCBI non-redundant database (NR) [29], Swiss-Prot, Cluster of Orthologous Groups database (COG) [30] and Kyoto Encyclopedia of Genes and Genome (KEGG) database [31], and were then aligned by BLASTN to nucleotide databases NT with E-value cutoff of 10^−6^. The best alignments were selected to annotate the unigenes. If the results of different databases contradicted with each other, a priority order of NR, Swiss-Prot, KEGG and COG was followed. Unigenes that could not be aligned to any database were annotated using ESTScan [32] to predict the protein coding regions.

Gene Ontology (GO) assignment of the unigenes was conducted using BLAST2GO software [33] with default parameters. To better understand the functions of the genes in the transcriptome, the online software package WEGO [34] was employed for functional classification of all the unigenes. The unigenes were also aligned to the COG database to predict and classify the potential functions. Pathway analysis of the unigenes were performed according to KEGG pathway database [31].

### Discovery of SNP (single nucleotide polymorphism) and SSR (simple sequence repeat) markers

The program SOAPsnp was used to screen putative SNPs with the unigenes as reference [35]. The raw reads from each fish were mapped onto the reference. The sequencing quality score was then recalibrated for SNP discovery with Bayes theorem. The identified SNPs were further filtered with more than 10 read depth and also with the quality score of more than 40 to obtain high quality SNP markers.

Microsatellite markers (SSRs) were detected using the software MicroSAtellite (MISA) [36] using unigenes as reference. The parameters were adjusted in order to identify mono-, di-, tri-, tetra-, penta-, and hexa-nucleotide motifs with a minimum of 12, 6, 5, 5, 4, and 4 repeats, respectively. Unigenes containing more than 150bp flanking regions on both sides of SSRs were retained for potential primer design using Primer3 [37].

### Identifying fast-evolving genes

To identify the orthologous genes between the two color morphs of coral trout, the predicted ORFs for each coral trout were subjected to BLAST searches using pairwise reciprocal best hits method with an E-value of 10^−20^ [38]. The protein sequences of each pair of putative orthologs were aligned using the program Muscle (v3.8.31) [39]. Then the nucleotide sequences of each pair of putative orthologs were transposed onto the corresponding protein alignment using the program PAL2NAL [40]. The alignments were further trimmed to remove gaps and poorly aligned sequences with T-Coffee (v11.00) [41].

After construction of CDS alignments for each pair of putative orthologous genes, the alignments of less than 20 codons were filtered out. The software KaKs_calculator (v2.0) [42] was employed to calculate the values of nonsynonymous substitution rate (Ka) and synonymous substitution rate (Ks) with the maximum-likelihood (ML) method [43]. The sequencing errors were suggested to be equally distributed at the nonsynonymous and synonymous sites and therefore they were not expected to influence the results of such analyses [44]. Unreliable alignments with Ks > 2 and Ka/Ks > 3 and with Ks = 0 were removed from further analysis [45]. The fast-evolving genes were defined as the genes with the top 5% Ka and Ka/Ks values [46]. The fast-evolving genes obtained were further enriched and annotated against the KEGG database [31].

### Identifying candidate genes involved in skin color and pigmentation

In order to identify the candidate genes that might be involved in the functions of skin color and pigmentation, the two transcriptome data sets were further searched by BLAST annotation and with the following GO terms including skin development (GO:0043588), pigmentation (GO:0043473), cellular pigmentation (GO:0033059) and developmental pigmentation (GO:0048066). The identified candidate genes were further studied with gene expression profiles by estimating the expected number of fragments per kilobase of transcript sequence per million base pairs sequenced (FPKM) [27].

## Results

### Transcriptome sequencing and assembly

In total, 66260084 and 67268086 raw reads were generated from the HiSeq^TM^2000 platform for black and red common coral trout, respectively. After filtering for quality control, 55162966 and 54588952 clean reads for the above two morphotypes were obtained, respectively, where 92.99% and 92.67% high-quality reads were separately used for transcriptome assembly (Table 1).

**Table 1:** Summary statistics of the paired-end Illumina sequencing of two common coral trout transcriptomes.

	Black trout	Red trout
Raw reads (n)	66 260 084	67 268 086
Clean reads (n)	55 162 966	54 588 952
Bases (bp)	4 964 666 940	4 913 005 680
Q20 (%)	92.99	92.37
GC content (%)	48.81	49.37

**Fig 1:**
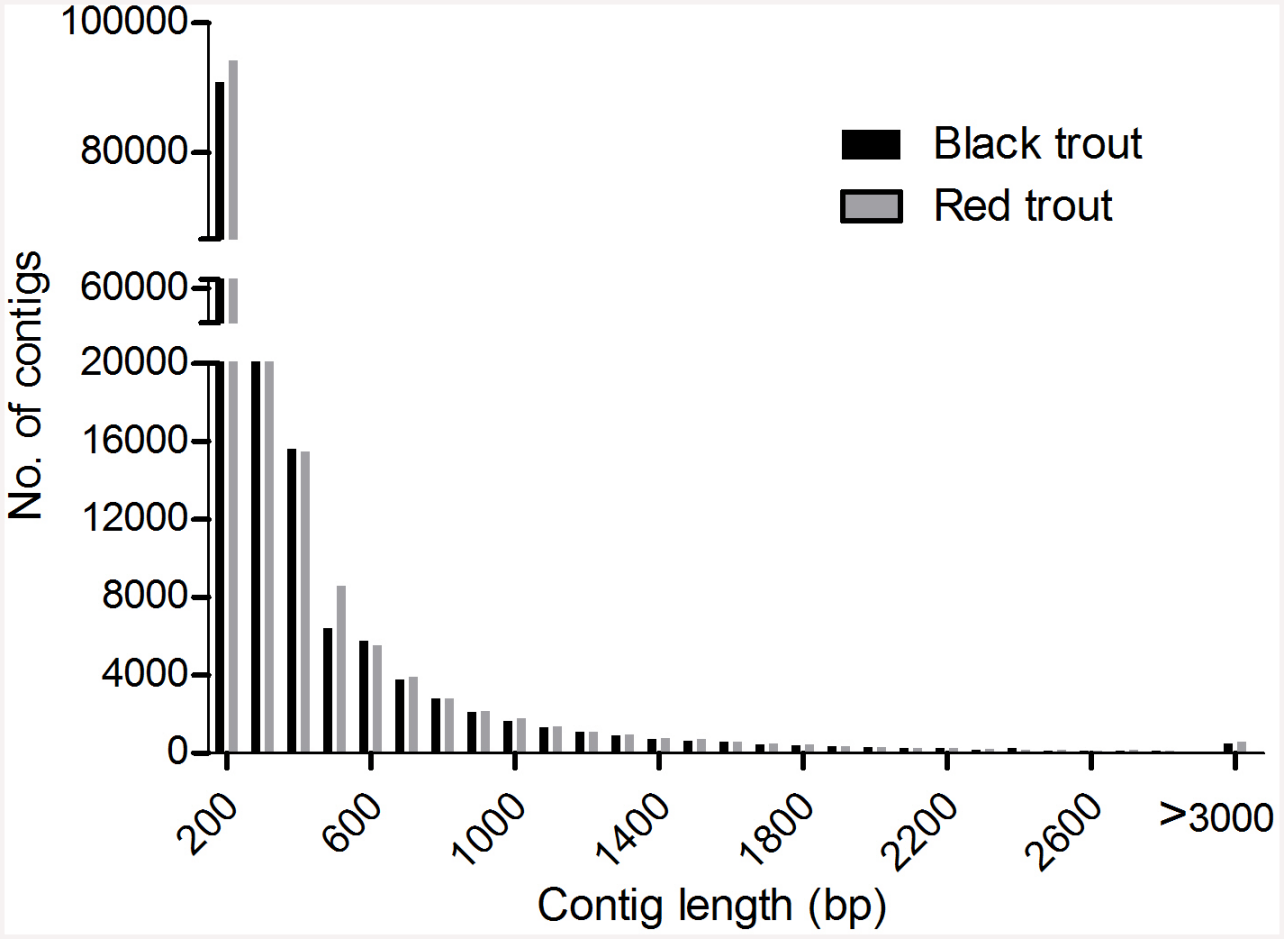
Contig length distribution of black and red common coral trout transcriptomes.

Trinity assembly produced 165264 and 171934 contigs with N50 lengths of 474 and 478 bp for black and red coral trout, respectively (Fig 1). BLAST analysis revealed 95367 and 99424 unigenes with N50 lengths of 796 and 816 bp, respectively for black and red coral trout (Table 2). The number of singletons for black and red coral trout was 81679 and 81889, respectively, with a consensus number of 70053 (Table 2).

**Table 2:** Summary statistics of de novo assembly results of two common coral trout transcriptomes.

	Black trout	Red trout	Consensus
No. contigs (n)	165 264	171 934	N.A.
Total contig length (bp)	52 653 758	54 942 263	N.A.
N50 for contigs (bp)	474	478	N.A.
No. unigenes (n)	95 367	99 424	88 813
N50 for unigenes (bp)	796	816	1 027
No. singletons (n)	81 679	81 889	70 053

### Functional annotation

Functional annotation of the whole unigene dataset of 88813 showed that 47585 (53.58%), 42181 (47.49%), 34110 (38.41%), 13442 (15.15%) and 30570 (34.42%) were positively matched to the NR, Swiss-Prot, COG, KEGG and Gene Ontology databases, respectively, which in total annotated 57756 unigenes (65.03%). The unigenes without BLAST hits were converted into deductive peptide sequences using ESTScan for CDS prediction and 1099 were suggested to be protein coding genes. Altogether, 48719 unigenes were classified as protein coding genes in the two transcriptomes, while the remaining were possibly the untranslated fragments of protein coding genes and non-coding RNA.

BLAST against NR database revealed that 66.83% and 10.43% of the unigenes matched to annotations of *Oreochromis niloticus* and *Tetraodon nigroviridis*, respectively, while the others were identified within the reference protein databases of *Danio rerio*, *Dicentrarchus labrax*, *Anoplopoma fimbria*, *Epinephelus coioides*, *Salmo salar* and the other species with mapping rates of less than 5% (S1 Fig). The 30570 unigenes with homology to NR database were assigned into 59 GO groups, which were organized into three major categories including Biological process, Cellular component and Molecular function (S2 Fig). The COG classification of 13442 unigenes were clustered into 25 functional categories, among which ‘general function prediction only’ was the largest group (6126 unigenes, 45.57%), followed by ‘Transcription’ (3062 unigenes, 22.78%), ‘replication, recombination and repair’ (2900 unigenes, 21.57%), ‘translation, ribosomal structure and biogenesis’ (2697 unigenes, 20.06%) and ‘cell cycle control, cell division, chromosome partitioning’ (2479 unigenes, 18.44%). The ‘nuclear structures’ (11 unigenes) and ‘extracellular structures’ (40 unigenes) had the least representations, of less than 1%, in the whole transcriptome (S3 Fig). KEGG pathway classification of the unigenes showed that 34110 unigenes were mapped to 259 pathways (S1 Table). The top five enriched pathways with the largest numbers of sequences were metabolic pathway, regulation of actin cytoskeleton, pathways in cancer, focal adhesion, and MAP kinase signaling pathways (MAPK) (S1 Table).

### SSRs and SNPs detection

**Fig 2:**
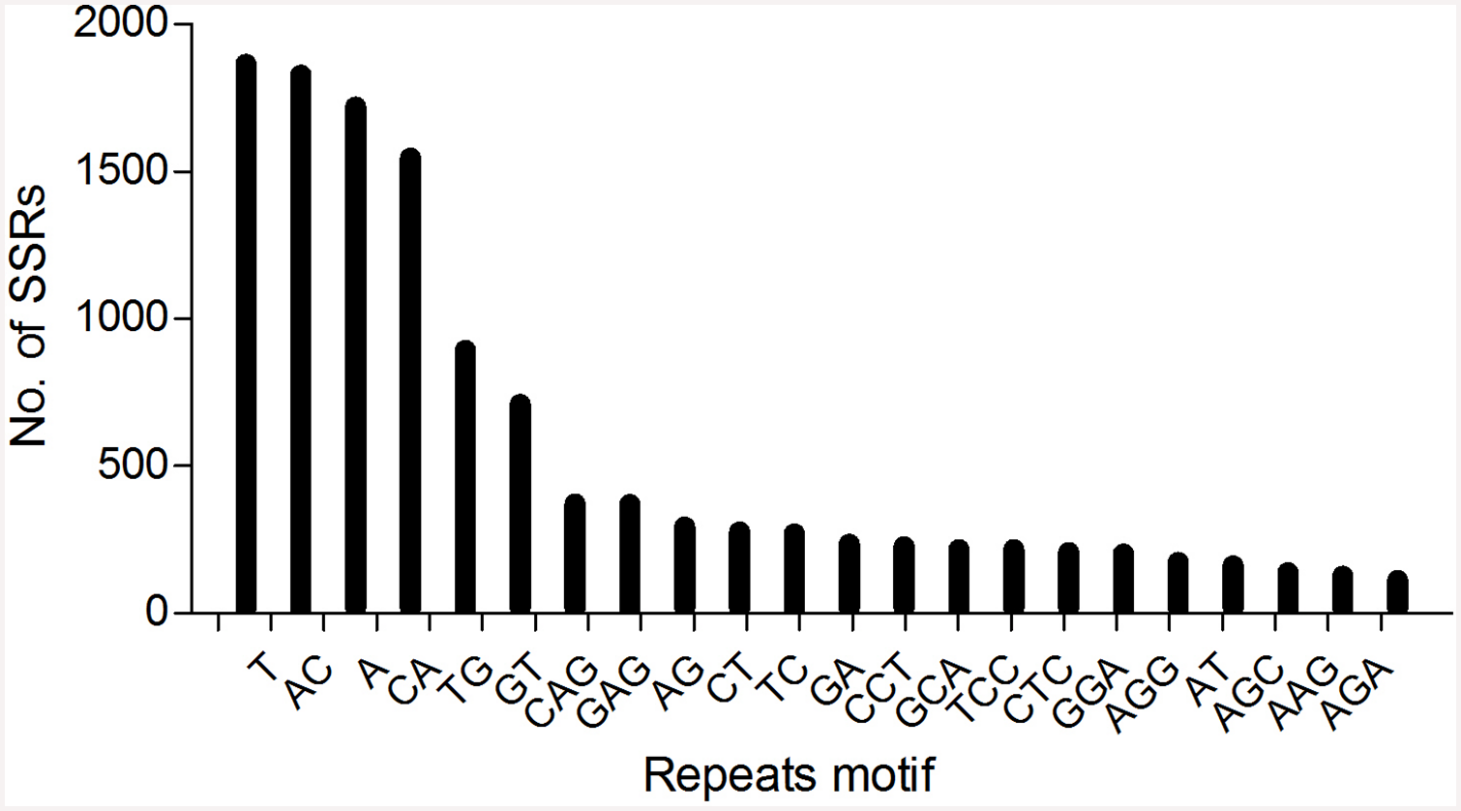
Summary of microsatellites repeat motifs and its richness across whole transcriptome of common coral trout.

In total, 14649 SSRs including 13431 with simple repeats and 1218 with compound form were identified in 11770 unique sequences, in which 2167 contained more than one SSR. Among these SSRs, di-nucleotide repeats were the most common SSRs with a number of 6332 accounting for 43.22% of the total SSRs, while tri-, mono-, quad-, penta- and hexa-repeats had numbers of 4020 (27.44%), 3716 (25.37%), 310 (2.12%), 153 (1.04%) and 118 (0.81%), respectively. Excluding the mono-repeats motif A and T flanking the 3’-end of CDS, the most common repeats motifs were the AC and TG, while the tri-repeats motif CAG and GAG were more common than the other di-repeats motifs (Fig 2). Excluding mono-repeats motifs, 95.4% of SSRs had a repeat number of no more than 10 (S4 Fig). After the filtering processes of flanking fragments and primer design, 8989 pairs of primers were developed for amplification of potential SSRs (S2 Table).

**Fig 3:**
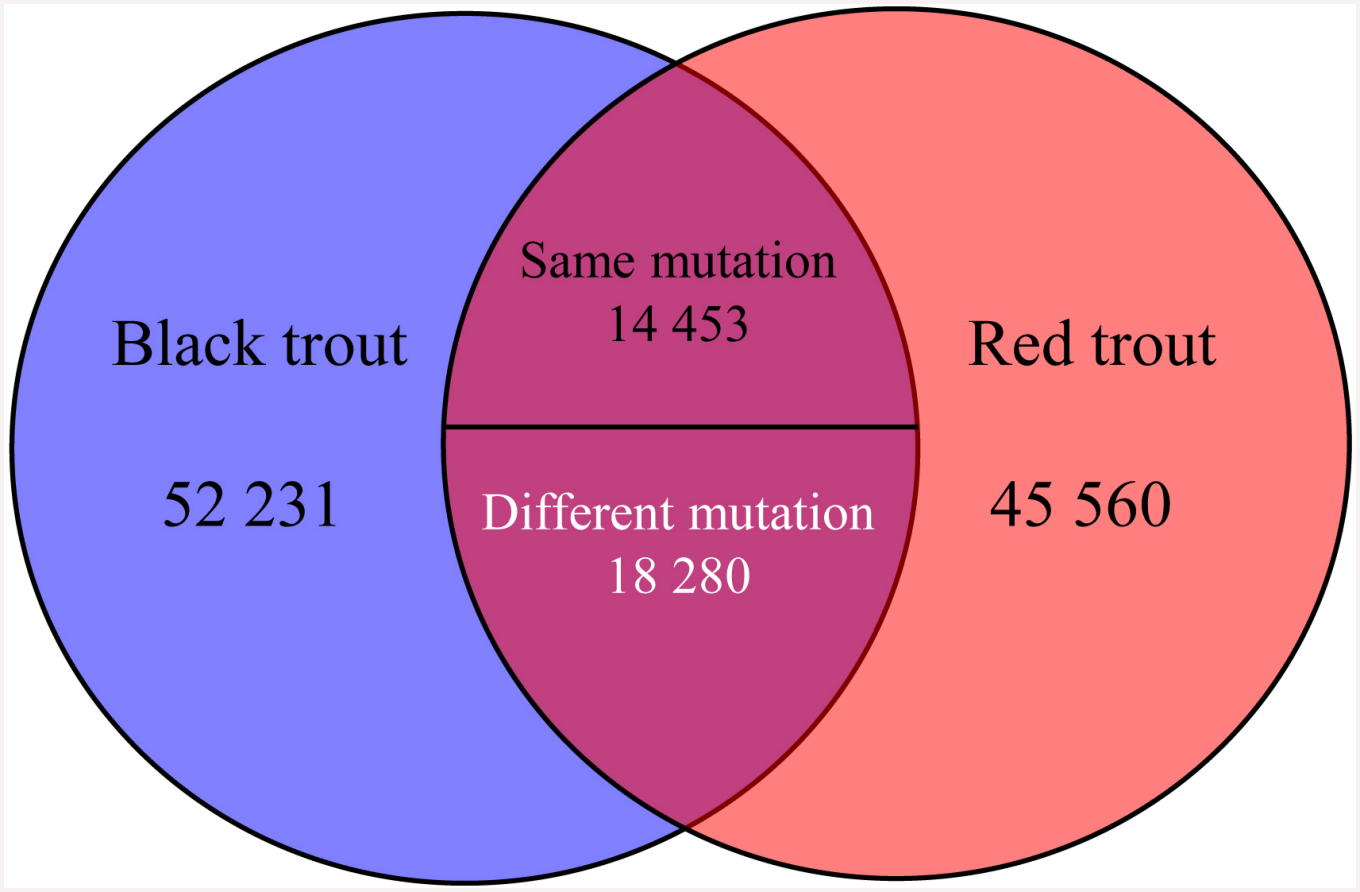
Venn diagram showing the difference and overlap in terms of the number of putative SNPs for the transcriptome of black and red common coral trout.

For SNPs discovery, a total of 130524 putative SNPs were identified across the whole trancriptome data sets (S3 Table). There were 84964 and 78293 putative SNPs including 60332 (71.01%) and 54913 (70.14%) transitions (A-G and C-T), and 24632 (28.99%) and 23380 (29.86%) transversions (A-C, A-T, C-G and G-T) within black and red trout, respectively (S5 Fig). The frequencies of transitions and transversions between black and red trout showed little difference (S5 Fig). The transcriptome-wide transition/transversion ratio within black (2.45) and red trout (2.35) was also similar. For the shared 32733 putative SNPs with same position between black and red trout, 14453 (44.15%) showed the same mutation patterns while 18280 (55.85%) had different mutation patterns (Fig 3 & S4 Table). After filtering out the putative SNPs with flanking regions of less than 60 bp, 106587 were obtained that can be used for future high-throughput SNP assay design (S3 Table).

### Identification of candidate genes under positive selection

**Fig 4:**
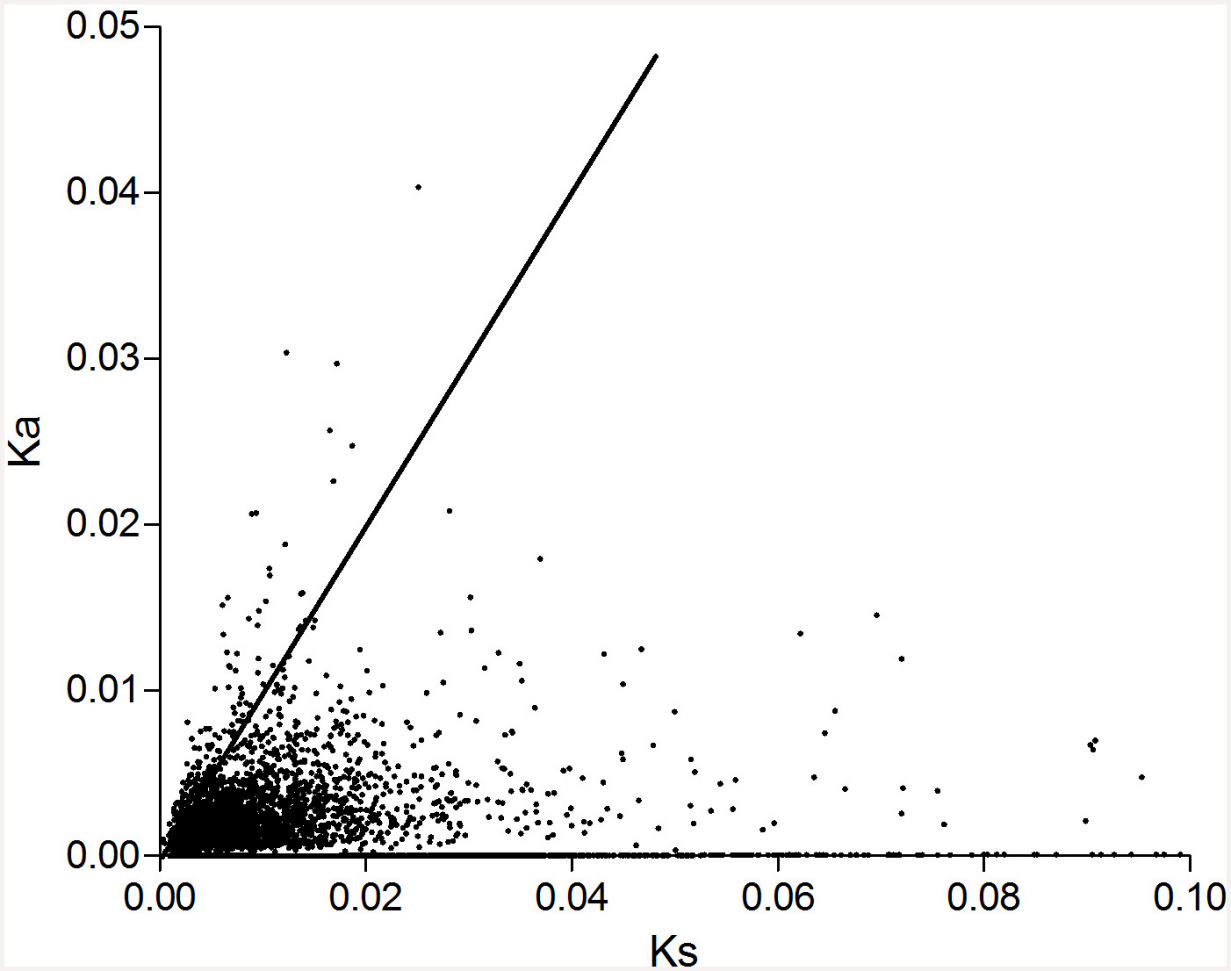
Plotting of the ratio of Ka/Ks of putative orthologous genes between black and red common coral trout, where the line indicates Ka/Ks = 1.

Sequence BLAST searches assigned 12403 pairs of putative orthologous genes between black and red trout, which had suitable sequence length and number of mutations for estimation of molecular evolution, and also could be calculated with Ka, Ks and Ka/Ks values by the program KaKs Calculator. The mean value of Ka, Ks, and Ka/Ks for the whole data set was 0.001, 0.011 and 0.086, respectively. In total, 165 pairs of orthologous genes had Ka/Ks > 1, within which 12 showed Ka/Ks > 2 (Fig 4 & S5 Table). These genes were mapped to the KEGG database to identify enriched biological signaling pathways. Interestingly, we found several pathways were significantly related to viral infection, e.g. herpes simplex infection, viral myocarditis, HTLV-I infection and viral carcinogenesis. Moreover, these candidate genes under positive selection were also enriched in the pathways of immune responses, such as lysosome, ECM-receptor interaction, platelet activation, NF-kappa B signaling pathway and Toll-like receptor signaling pathway. Finally, we also found some basic biological pathways including cell adhesion, metabolic pathways, RNA transport, cAMP signaling pathway and so on (S6 Table).

Among 120403 orthologous genes, 936 were identified as fast-evolving genes ranking top 5% of Ka and/or Ka/Ks values. Further analysis of these genes by mapping to the KEGG pathways revealed 87 pathways with each including at least 5 fast-evolving genes (S7 Table). These pathways cover a wide range of biological functions, including basic metabolic pathways, cell cycle, growth and development, energy metabolism, cytoskeleton and cell adhesion, microbial infection, immune and stress responses, calcium signaling pathway and hypoxia (HIF-1 signaling pathway) (S7 Table).

### Identifying candidate genes involved in skin color and pigmentation

Candidate gene enrichment analysis identified 38 candidate genes involved in the processes of skin color and pigmentation from the two transcriptome data sets, among which 34 showed signals of being differentially expressed between black and red trout (Table 3). Interestingly, nine genes were revealed to be specifically expressed in black trout, while only one was found uniquely expressed in red trout (Table 3).

**Table 3:** Expression and annotation of candidate genes involved in skin color and pigmentation between black and red common coral trout.

Gene_ID	FPKM Black trout	FPKM Red trout	Fold change (log2)	Annotation
CL4641.Contig1	0.000	22.314	14.446	Bifunctional methylenetetrahydrofolate dehydrogenase/ precursor
CL4641.Contig2	0.000	10.430	13.348	Bifunctional methylenetetrahydrofolate dehydrogenase
CL4045.Contig2	0.000	9.946	13.280	Unnamed protein product
Unigene59055	0.000	8.618	13.073	Serine hydroxymethyltransferase
CL6418.Contig3	0.000	8.074	12.979	Unnamed protein product
CL2910.Contig2	0.000	5.772	12.495	TCP1-beta
CL7006.Contig2	0.000	5.036	12.298	5-aminolevulinate synthase
CL1293.Contig2	0.000	5.005	12.289	V-type proton ATPase subunit S1-like
CL6316.Contig2	0.000	3.816	11.898	Porphobilinogen deaminase-like isoform 2
Unigene63365	0.129	7.517	5.865	Putative 14-3-3 protein
CL5424.Contig1	3.534	136.183	5.268	5-aminolevulinate synthase, erythroid-specific
CL2159.Contig2	0.133	2.129	3.996	Flavin reductase-like
CL3964.Contig1	1.156	7.832	2.761	Rab-3A-interacting protein-like
CL4641.Contig3	0.359	2.382	2.730	Bifunctional methylenetetrahydrofolate dehydrogenase/cyclohydrolase
CL5424.Contig2	6.336	40.144	2.664	5-aminolevulinate synthase, erythroid-specific
CL263.Contig1	0.934	5.027	2.429	Adenosine deaminase
Unigene61602	1.397	6.643	2.250	Homeodomain-interacting protein kinase 2
CL5425.Contig1	2.028	7.175	1.823	Dual specificity mitogen-activated protein kinase kinase 1-like isoform 2
Unigene25109	4.911	14.484	1.560	Homeodomain-interacting protein kinase 2
CL4718.Contig1	10.243	25.545	1.318	Endoplasmin-like
Unigene12593	2.381	5.780	1.280	Trifunctional purine biosynthetic protein adenosine-3-like
CL1174.Contig2	17.688	38.970	1.140	Protein disulfide-isomerase-like
Unigene4585	6.257	13.747	1.136	Mediator of RNA polymerase II transcription subunit 12-like
CL5300.Contig1	3.277	6.883	1.071	Deaminase-like protein
CL4861.Contig1	2.503	5.108	1.029	Calnexin-like
CL3531.Contig1	1.439	0.000	-10.491	Formimidoyltransferase-cyclodeaminase-like
CL5259.Contig2	7.013	0.193	-5.186	Protein disulfide-isomerase A3
CL2569.Contig1	4.666	0.583	-3.002	Unnamed protein product
CL3964.Contig2	5.873	1.013	-2.535	Rab-3A-interacting protein-like
CL4718.Contig2	39.828	13.877	-1.521	Endoplasmin-like
CL5670.Contig3	7.336	3.414	-1.103	Phenylalanine-4-hydroxylase
CL1522.Contig1	12.803	6.100	-1.070	Macrophage colony stimulating factor receptor
Unigene9810	11.226	5.376	-1.062	Molybdenum cofactor biosynthesis protein 1-like
Unigene24574	9.797	4.792	-1.032	Bardet-Biedl syndrome 5 protein homolog

## Discussion

With the development of NGS technologies, genomic resources have been greatly increased in non-model fish species. However, in coral reef fish species, there were still little genomic resources available. These resources were all developed in the Serranidae family, *Epinephelus* genus [47, 48]. Here, we developed the first transcriptome resources of common coral trout for the genus *Plectropomus* of the same family, Serranidae, by sequencing two color morphs of this coral trout using the Illumina Hiseq^TM^2000 platform. The transcriptomes were from many types of tissue and therefore can greatly represent the whole transcriptome of this species.

Over 66 million paired-end reads were produced for each color morph of coral trout and 88813 unigenes were assembled with N50 of about 800 bp in length (Tables 1 & 2). These results suggest a good coverage of transcriptome sequencing in fish species of normal genome size [49]. We also identified a number of 70053 unigenes that were consensus genes between the two color morphs of common coral trout; these consensus genes are potentially useful for future comparative trancriptome analysis [50–52]. Annotation of the unigenes showed that 48719 unigenes were protein coding genes, which is similar with the results obtained in non-model fish species using *de novo* assembly of transcriptomes [53, 54], but is higher than that of the model fish species with less than 30000 protein coding genes, such as zebrafish and Nile tilapia [55, 56]. These differences are largely due to the great complexity of *de novo* assembly of transcriptome and the comprehensively complicated structure of the whole genome of common coral trout. Moreover, over 65% unigenes were successfully annotated in this study, which is much higher than that of the other studies in non-model fish species. BLAST annotation revealed that over 66% of unigenes could match to the annotation of Nile tilapia, suggesting a close relationship between coral trout and tilapia (S1 Fig). In addition, we found little difference in terms of transcriptome profile between black and red common coral trout (Fig 1 & Table 2). It should be noted that there were also many unigenes without any BLAST hit. These sequences might represent novel genes that have not been included in the annotated protein databases.

Trancriptome data sets are widely used for the development of genomic tools including genome-wide microsatellite and SNP markers that can be used in population genetic studies [25, 49, 57]. Here, we identified 14649 microsatellites and developed 8989 pairs of primers for amplification of these loci (S2 Table). Although the primers were not tested in laboratory and also some of the primers might not be annealed to the genomic DNA sequences due to locating on the spanning boundaries of exons, the development of microsatellite markers would be an important and valuable genomic resource for future population genetic studies of this species or the other coral trout species. In terms of SNP discovery, 130524 putative SNPs were detected in the whole trancriptome data sets and 106587 had enough flanking sequences for future high-throughput SNP assay design (S3 Table). Importantly, we identified 18280 putative SNPs that showed different patterns of nucleotide mutation (S4 Table). This type of SNPs provides very valuable genomic resources for population genetic studies, genetic mapping and screening the mutations underlying the ecological speciation and divergence between and among coral trout species [58, 59].

The value of Ka/Ks has also been widely used to estimate the evolving rate and mode of selection of genes [46, 60]. Genes with Ka/Ks > 1 are suggested to be under putatively positive selection, while genes with Ka/Ks < 1 are under putatively purifying selection [42]. We identified 165 pairs of orthologous genes with Ka/Ks > 1 (Fig 4 & S5 Table). These genes were involved in various biological functions, suggesting these pathways possibly suffer from selective pressure during the process of speciation and divergence between the two color morphs and therefore play critical roles in biological variations of coral trout species [60]. Interestingly, we found that several pathways were associated with virus infection and immune responses, such as herpes simplex infection, viral myocarditis, HTLV-I infection, viral carcinogenesis, lysosome, ECM-receptor interaction, platelet activation, influenza A, NF-kappa B signaling pathway and Toll-like receptor signaling pathway (S6 Table). These results indicate virus infection and/or host-pathogen interactions likely have important roles in driving ecological speciation of common trout species [61, 62]. In addition, we also found 936 fast-evolving genes between the two morphs of common coral trout. These genes also have a wide range of biological functions, suggesting their critical roles in contributing to the genetic divergence between the two morphs of common coral trout [46].

Candidate gene analysis found that 34 of 38 genes that are likely responsible for different skin colors and pigmentation between black and red coral trout were differentially expressed between the two color morphs (Table 3). Interestingly, 10 genes were specifically expressed in either black or red common coral trout. Many of these genes have been studied and found to play important roles in coloration and pigmentation of different species. Previous studies have revealed that Serine hydroxymethyltransferase (SHMT) is associated with green pigments [63], chaperonin containing protein 1 (TCP1) is responsible for the plumage coloration of flycatchers [64], 5-aminolevulinate synthase (ALAS) is the first enzyme of heme biosynthesis [65] and V-type proton ATPase subunit S1 (ATP6AP1) regulates the eye pigmentation of zebrafish [66]. Interestingly, porphobilinogen deaminase gene determines the camouflage patterning in maize [67]. Besides those, many other genes identified in this study were also revealed to have critical roles in coloration and pigmentation of plants and animals. However, it should be noted that we used pooled samples for in-silico detection of the differentially expressed genes. Without further experimental verification, it is difficult to accurately quantify the expression levels of these genes. Thus some genes, in particular those showing low levels of differential expression, might be false positives. Nevertheless, the 10 specifically expressed genes between the two studied color morphs are seldom influenced by such pooling strategy. Future study on the gene expression profiles and functions of these genes will help a lot in understanding the mechanisms of ecological divergence and speciation of skin color and pigmentation among coral trout species. Here, the value of this study is primarily to identify the possible genes for further study.

Above all, this study provides very valuable genomic resources in terms of gene identification, genomic markers discovery and candidate gene screening for investigating the genomics, population genetics, ecological speciation and evolution of coral trout species.

### Data accessibility

Raw sequence reads have been submitted to the Sequence Read Archive of NCBI (Accession no.SRR1743130 & SRR1743132).

## Supporting Information

S1 Fig The percentage of the whole transcriptome of black and red common coral trout that mapped to different fish species in NR annotation. (JPG)

S2 Fig The GO enrichments of the whole transcriptome of black and red common coral trout using the program WEGO. (JPG)

S3 Fig The COG function classification of the whole transcriptome of black and red common coral trout. (JPG)

S4 Fig The richness and distribution of six different types of repeat motifs of microsatellites across whole transcriptome of common coral trout. (JPG)

S5 Fig The percentage of transitions and transversions of putative SNPs identified from black and red common coral trout. (JPG)

S1 Table Enrichment of 259 KEGG pathways for the whole transcriptome of black and red common coral trout. (XLS)

S2 Table 8989 primers and parameters designed for amplification of putative microsatellites of black and red common coral trout. (XLS)

S3 Table All putative SNPs (130 524) identified in the transcriptomes of black and red common coral trout. (XLS)

S4 Table 18280 putative SNPs showed different at the same chromosome positions between black and red common coral trout. (XLS)

S5 Table Candidate genes under positive selection with Ka/Ks more than one between black and red common coral trout. (XLSX)

S6 Table KEGG pathways enriched using candidate genes under positive selection and the number of genes involved in each pathway. (XLSX)

S7 Table KEGG pathways enriched using fast-evolving genes and number of genes involved in each pathway. (XLSX)
