# Supplementary figures and images for: In Silico Comparative Transcriptome Analysis of Two Color Morphs of the Common Coral Trout (Plectropomus Leopardus)
Source: PLoS One. 2015 Dec 29;10(12):e0145868. doi: 10.1371/journal.pone.0145868 (PMC4700983; doi:10.1371/journal.pone.0145868)

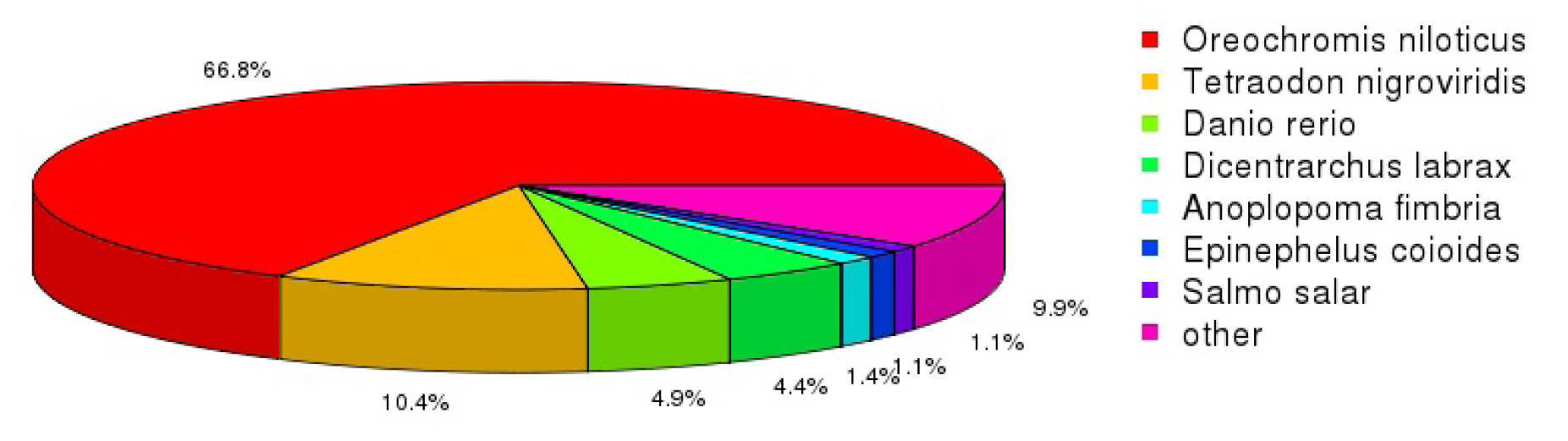

Supplement: S1 Fig — (JPG) [file pone.0145868.s001.jpg]

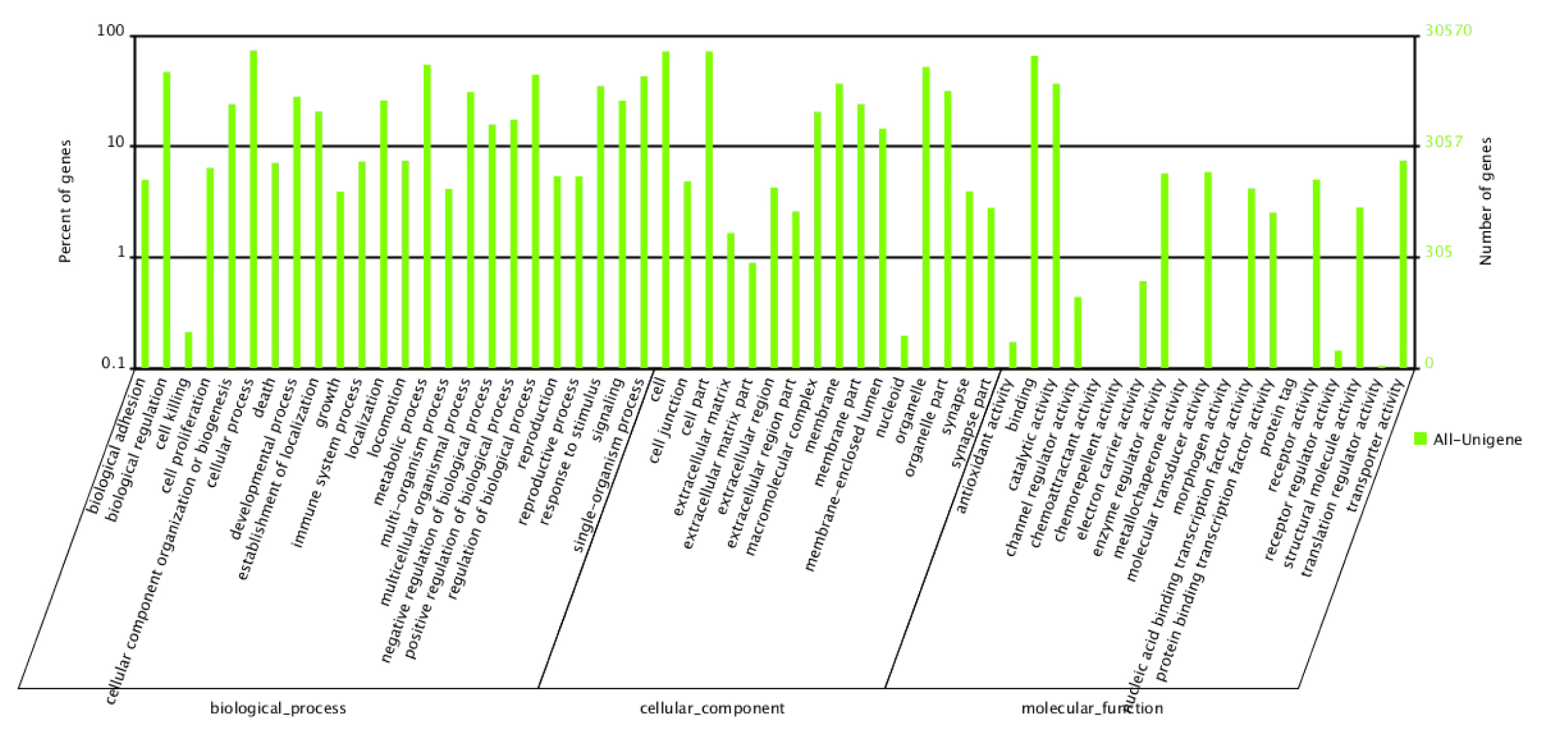

Supplement: S2 Fig — (JPG) [file pone.0145868.s002.jpg]

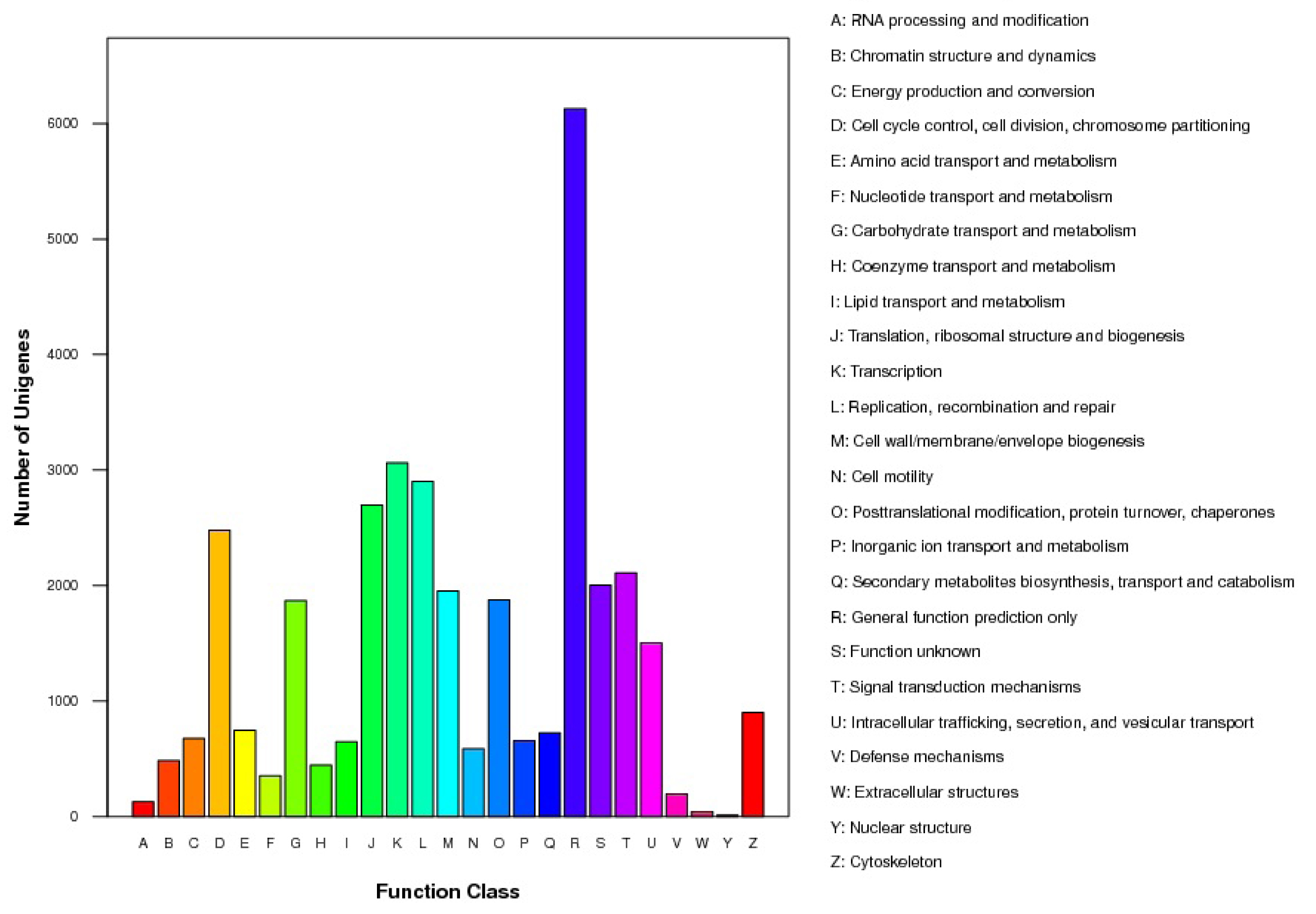

Supplement: S3 Fig — (JPG) [file pone.0145868.s003.jpg]

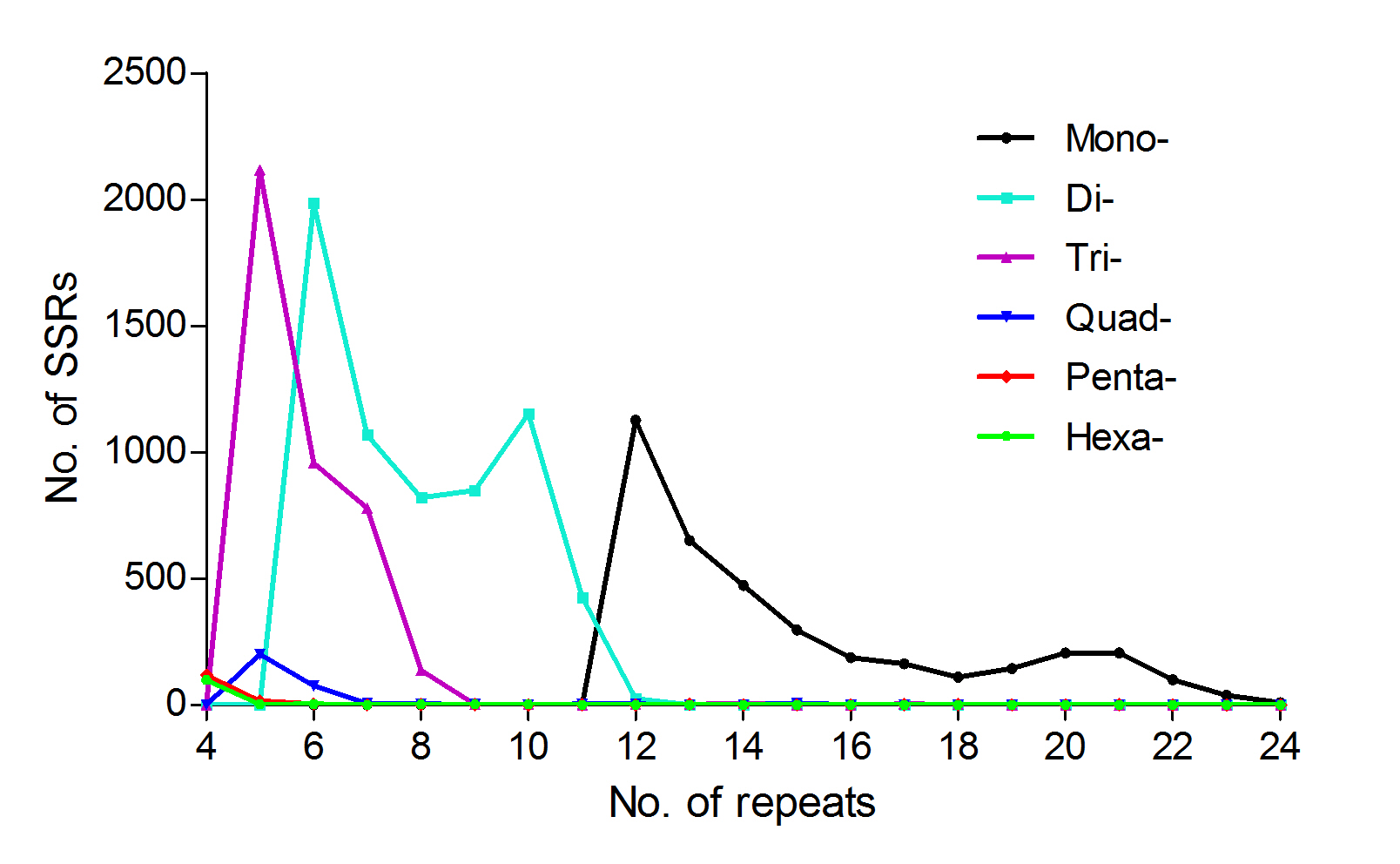

Supplement: S4 Fig — (JPG) [file pone.0145868.s004.jpg]

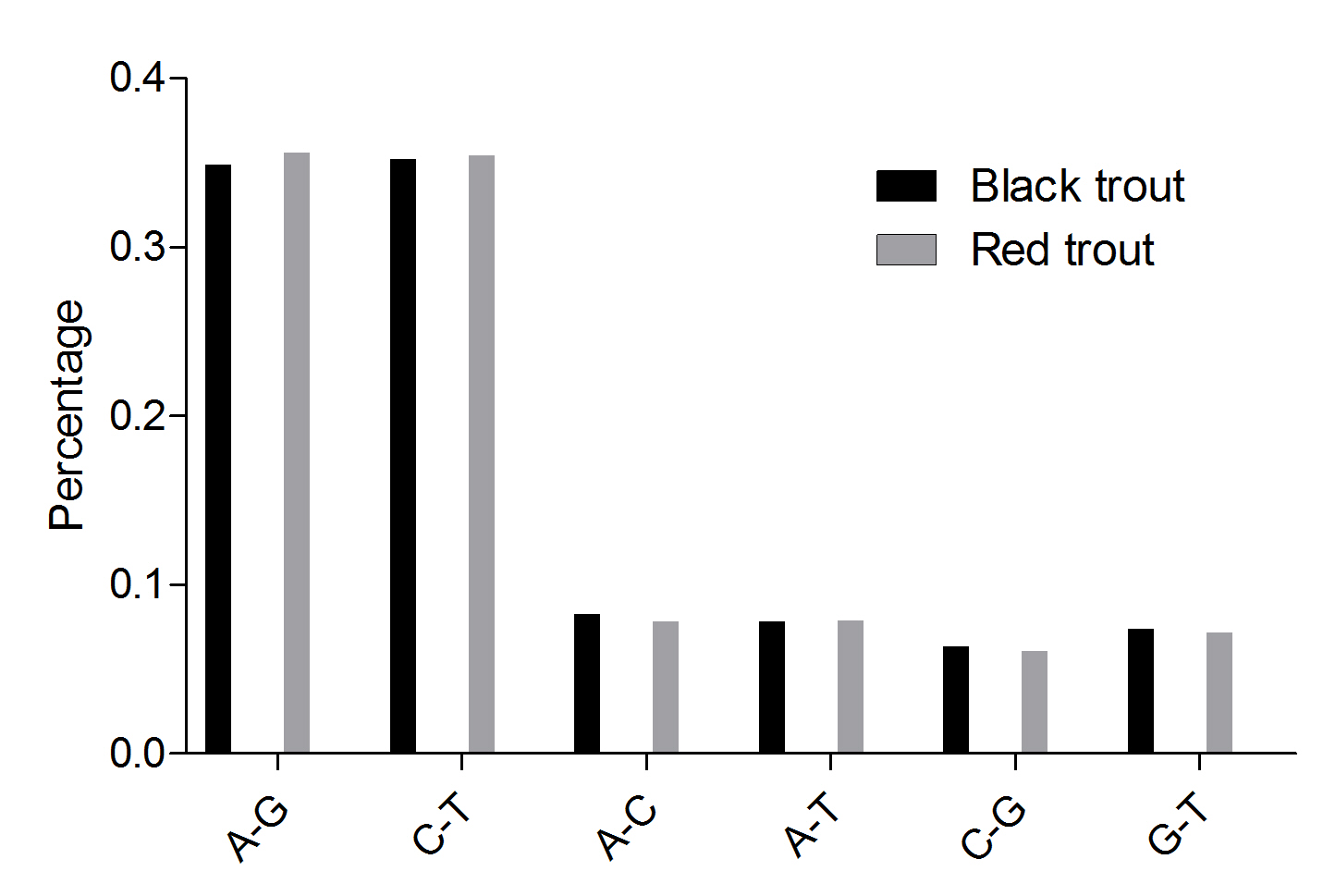

Supplement: S5 Fig — (JPG) [file pone.0145868.s005.jpg]
